# Research progress on zero-time exercise interventions in patients with chronic diseases: a narrative review

**DOI:** 10.3389/fpubh.2025.1643407

**Published:** 2025-11-07

**Authors:** Guilan Ban, Chaxiang Li, Yue Jin

**Affiliations:** 1 The First College of Clinical Medical Science, China Three Gorges University/Yichang Central People's Hospital, Yichang, Hubei, China; 2 College of Medicine and Health Sciences, China Three Gorges University, Yichang, Hubei, China

**Keywords:** zero-time exercise, chronic diseases, physical activity, health behavior, exercise prescription

## Abstract

Traditional exercise programs are often challenging for patients with chronic diseases due to time constraints, physical limitations, and other barriers. Zero-time exercise (ZETx) is an innovative approach that requires no additional time, equipment, or financial investment, making it particularly suitable for these patients. This narrative review aims to explore the conceptual framework, theoretical foundations, and health benefits of ZETx, as well as its acceptability and adherence among chronic disease patients. We conducted a comprehensive search in electronic databases (PubMed, Embase, Web of Science, Cochrane Library) using relevant search terms related to ZETx. Studies published in English and focusing on ZETx interventions were included. The search spanned from the inception of the databases to July 2025. ZETx interventions have demonstrated significant health benefits, including improvements in cardiorespiratory function, musculoskeletal health, cognitive function, and metabolic parameters. Additionally, ZETx has been found to be highly acceptable and feasible among chronic disease patients, with adherence rates exceeding those of traditional exercise programs. Future research should focus on standardizing exercise prescriptions, leveraging technological innovations such as wearable devices and virtual reality, and developing multidisciplinary intervention models. This approach has the potential to transform current practice paradigms in chronic disease management by providing a novel pathway for improving cardiovascular health, metabolic capacity, and overall wellbeing.

## 1 Introduction

With rapid socioeconomic development and lifestyle changes, health issues caused by sedentary behavior have become increasingly prominent (1). Sedentary behavior is closely associated with various chronic diseases, such as cardiovascular diseases, obesity, diabetes, and depression, posing a major global public health challenge (2). According to the World Health Organization (WHO), over 1.4 billion adults worldwide lack sufficient physical activity, and their health status requires urgent improvement (3). The latest WHO guidelines on physical activity and sedentary behavior emphasize that “every move counts” and recommend reducing sedentary time, as replacing it with any intensity of activity can yield benefits (4). Lack of time is the most common barrier preventing individuals from initiating or maintaining exercise, followed by financial costs, limited access to facilities, and a dislike for vigorous exercise (5). Patients with chronic diseases often face additional challenges, such as physical limitations, contraindications to exercise, or low exercise tolerance, making structured exercise programs difficult to adopt. Occasional physical activity may be more feasible and appealing than structured exercise, as it requires minimal time commitment and equipment (6). ZETx, as an emerging intervention strategy, overcomes the traditional limitations of time, space, and equipment, offering an innovative solution for physically inactive individuals with chronic diseases (7). In recent years, China has vigorously promoted national fitness and the Healthy China initiative. The Healthy China Action (2019–2030) and the National Fitness Plan (2021–2025) explicitly advocate for widespread participation in physical activity across all demographics, integrating fitness with health promotion and providing policy support for the application of zero-time exercise among chronic disease patients (8, 9). Prior reviews on incidental or fragmented physical activity have largely focused on general populations or broad activity categories, without specifically targeting chronic disease patients or emphasizing the “zero-time” principle—i.e., no extra time dedicated to exercise (10). This review advances the field by concentrating on ZETx as a tailored intervention for chronic disease management, synthesizing evidence on its efficacy, acceptability, and feasibility in this specific population. By doing so, it addresses the critical gap in translating ZETx concepts into practical strategies for patients facing significant barriers to traditional exercise.

## 2 Methodology

### 2.1 Literature search strategy

To ensure a comprehensive overview of the current evidence on ZETx, a structured literature search and selection process was conducted. This review adopted a narrative synthesis approach, aiming to summarize and critically appraise the existing literature rather than to perform a quantitative meta-analysis. The present study conducted a search in electronic databases (PubMed, Embase, Web of Science, Cochrane Library) using appropriate search terms related to zero-time exercise. The search terms included “zero time exercise, “exercise snack,” “exercise snacks,” “exercise snacking,” “incidental physical activity,” “vigorous intermittent lifestyle physical activity,” “micro exercise,” “Snacktivity,” and “short bouts of exercise.” The titles and abstracts were initially screened to identify potentially relevant studies. The search spanned from the inception of the databases to July 2025. The inclusion criteria were studies focusing on zero-time exercise and published in English. The exclusion criteria included studies not related to zero-time exercise, studies published in languages other than English, and studies without full-text availability. Given the heterogeneity in study designs, populations, and outcome measures, a narrative synthesis method was employed. The extracted information from the literature included authors, study population, intervention, duration of intervention, and outcome measures (Table 1).

**Table 1:** Basic characteristics of included literatures.

**References**	**Intervention type**	**Population**	**Duration**	**Main outcome**
Yeung et al. (14)	Exercises ranging from simple stretching to resistance training, which can be performed while sitting, standing, or walking	Patients with insomnia	24 weeks	Insomnia severity, fatigue, quality of life, physical activity, adherence
Zhou et al. (57)	Stair climbing sprint exercise (60 steps each time, 6 times per day, with an interval of >1 h between each time, 4 times per week)	Obese adults	12 weeks	Plasma metabolomics
Thirunavukkarasu et al. (21)	2-min activity of climbing two floors of stairs every 30 min for a continuous 2 h	Sedentary adults	2 hours	Blood glucose level
Daley et al. (15)	Receiving zero-time exercise intervention, where participants perform 2–5 min of “snack-sized” exercises daily, such as stair climbing, short-duration gardening, going up and down stairs, walking in the park, rope skipping, etc., with a total weekly exercise time of ≥150 min	Sedentary adults	12 weeks	Adherence, physical activity
Jones et al. (10)	1 min of in-place running for 3 times a day, with an interval of at least 1 h between two exercise sessions, conducted 4 days a week	Sedentary office workers	4 weeks	Cognitive function
Yin et al. (16)	Stair climbing exercise with 3 sets of approximately 30 s each, 3 times a week, with an interval of >1 h between sets, for 6 consecutive weeks	Physically inactive college students	6 weeks	Adherence, quality of life, perceived stress
Yin et al. (23)	Each training session includes 3 × 30 s of stair climbing exercise, 3 times a week	Sedentary adults	6 weeks	Cardiopulmonary function
Brandt et al. (17)	10-min zero-time resistance exercises near the office, 5 days a week, such as squats, push-ups, lunges, wall sits, side bends, standing knee lifts, calf raises, and standing leg lifts	Sedentary female office workers	12 weeks	Muscle mass
Sun et al. (18)	SEM-based zero-time exercise intervention; zero-time exercises include shoulder shrugs, tilting the head up and down, lifting legs off the ground, and cycling in the air. Most of these exercises can be performed while standing, sitting, walking, lying down, watching TV, or waiting for transportation	Community-dwelling adults	3 months	Physical activity, mental health
Campbell et al. (58)	Breaking up sedentary time with 3-min light-intensity walking every 30 min for 7 hs	Sedentary patients with type 1 diabetes	Two 7-h periods	Average blood glucose level within 48 h
Yun et al. (59)	Short intermittent stair climbing exercise, including 3 min of warm-up, followed by 3 rounds of 20-s stair climbing with 2-min recovery periods in between, 5 times a week	Overweight and obese women	4 weeks	Cardiopulmonary function, body weight, BMI, waist circumference
Chan et al. (19)	Zero-time exercises of any type and duration, such as simple strength and endurance enhancement exercises, brisk walking, stair climbing, etc., with a gradual increase in intensity	Patients with coronary heart disease	12 weeks	Physical activity, feasibility
Fyfe et al. (20)	9-min home-based zero-time exercise performed once, twice, or three times a day (including sit-ups, squats, side lunges, calf raises, and stepping, with 1 min for each exercise and 1 min of rest between exercises)	Community-dwelling older adults	4 weeks	Feasibility, acceptability
Rafiei et al. (44)	“Snack-sized” fast stair climbing exercise of 15–30 s once per hour, conducted 8 times within 9 h	12 young healthy men and 11 adults with overweight/obesity	9 hours	Postprandial insulin, total non-esterified fatty acids, feasibility
Lai et al. (12)	Receiving “zero-time exercise” intervention, such as lifting heels while standing, lifting feet and legs off the ground while sitting, or stretching exercises	Low-income family members in Hong Kong	12 months	Physical activity, family communication, perceived health
Lai et al. (13)	Exercises ranging from simple stretching to resistance training, which can be performed while sitting, standing, or walking	Community service workers	3 months	Balance, muscle strength, physical activity
Little et al. (24)	3 rounds of 20-s all-out cycling sprints each time, 3 times a week, with 1–4 h of rest between rounds	Sedentary adults	6 weeks	Cardiopulmonary function
Yeung et al. (5)	Exercises ranging from simple stretching to resistance training, which can be performed while sitting, standing, or walking	Patients with insomnia	8 weeks	Insomnia Severity Index, grip strength, adherence, acceptability

## 3 Overview of zero-time exercise

### 3.1 Concept of zero-time exercise

ZETx is an innovative physical activity approach first proposed by the School of Public Health at The University of Hong Kong in 2016 to address the time-consuming and costly nature of traditional structured exercise (11). It is characterized by the “Three-Zero” principle—requiring no extra time, no financial cost, and no additional equipment—while fulfilling the “Three-A” conditions: it can be performed Anytime, Anywhere, by Anyone (12). ZETx is defined as a series of brief, low-to-moderate intensity physical activities that can be seamlessly integrated into daily routines without the need for dedicated exercise time or specialized equipment (13). These activities typically include simple stretching, resistance training that can be performed while sitting, standing, or walking, stair climbing, short-duration gardening, going up and down stairs, walking in the park, rope skipping, and other similar movements (12, 14–20). The key feature is that these exercises are designed to be easily incorporated into everyday activities, such as during breaks at work, while waiting for transportation, or while watching TV. The duration of ZETx activities is typically short, ranging from a few seconds to a few minutes per bout. For example, some studies have used 1-min in-place running sessions three times a day (10), while others have employed 2–5 min of “snack-sized” exercises daily, such as stair climbing or short-duration gardening, with a total weekly exercise time of ≥150 min (15). The intensity of ZETx is generally low to moderate, aiming to be accessible to individuals with varying levels of physical fitness and chronic disease severity. It is designed to be performed at a level that is comfortable and sustainable for the individual, without causing excessive fatigue or discomfort. The frequency of ZETx can vary widely, from several times a day to a few times a week, depending on the specific intervention and the individual's capabilities and preferences. Some studies have participants perform ZETx activities multiple times a day (e.g., every 30 min during an 8-h workday) (10, 21, 22), while others have a less frequent schedule, such as three times a week (16, 23, 24). Due to its adaptability, ZETx is particularly suitable for chronic disease patients, allowing them to personalize exercise intensity based on their condition (20). Through progressive and cumulative ZETx effects, ZETx can help improve physiological functions in patients with varying disease severities (7).

### 3.2 Theoretical foundation of zero-time exercise

ZETx is developed based on the Health Action Process Approach (HAPA) model, which addresses psychological and cognitive factors influencing behavior change, including risk perception, outcome expectancies, and self-efficacy (13). The model aims to modify participants' mindset by enhancing their self-efficacy and interest, thereby fostering a sense of positive achievement (25). The HAPA model bridges the intention-behavior gap through two distinct phases: the motivational phase and the volitional phase. The motivational phase involves risk perception, outcome expectancies, perceived self-efficacy, and intention formation (i.e., goal setting). The volitional phase encompasses action control, maintenance self-efficacy, and recovery self-efficacy (26). In ZETx interventions, chronic disease patients are first educated about the hazards of sedentary behavior (risk perception) and the health benefits of physical activity (outcome expectancies) (19). Then, individualized and achievable exercise goals are established (goal setting) to enhance participation motivation. During implementation, continuous action guidance and feedback support (action control) are provided to help patients overcome potential barriers while maintaining and restoring their self-efficacy (27). This comprehensive approach ensures the sustainability and effectiveness of the exercise intervention.

## 4 Health benefits of zero-time exercise

### 4.1 Cardiorespiratory function

As an independent risk factor for cardiovascular health, sedentary behavior can be effectively addressed through ZETx interventions. Studies have demonstrated that for chronic disease patients with prolonged physical inactivity, ZETx activities such as stair climbing and brisk walking significantly reduce adverse cardiovascular events and mortality rates (28, 29). Chan et al. (20) conducted a 12-week ZETx intervention incorporating smartphone monitoring in 139 patients with coronary artery disease (CAD). The intervention not only increased physical activity levels but also improved quality of life in CAD patients, providing a reference model for remote exercise management in this population. Mechanistically, ZETx enhances cardiorespiratory fitness by approximately 10–17% (30) through activation of cardiopulmonary function and promotion of blood circulation. These physiological adaptations play a crucial protective role in maintaining cardiovascular health among chronic disease patients.

### 4.2 Musculoskeletal system protection

Prolonged sedentary behavior contributes to decreased skeletal muscle mass, and ZETx offers an effective strategy to mitigate this deterioration. In contrast to conventional training methods, the low-dose ZETx approach not only enhances muscle strength in older adults individuals but also improves the flexibility and feasibility of exercise programs (31). Evidence from a randomized controlled trial indicates that short-term ZETx interventions can significantly increase muscle strength, alleviate musculoskeletal pain symptoms, prevent progressive declines in work capacity, and optimize psychosocial factors in work environments (32). For example, a study examining intermittent resistance exercise in community-dwelling older adults demonstrated a 32% improvement in 1-min sit-to-stand test performance following the intervention. Notably, some participants achieved Short Physical Performance Battery (SPPB) scores exceeding 9 points (with baseline inclusion criteria of 3–8 points), reaching clinically significant levels of muscle function improvement (33). Further supporting these findings, Perkin et al. implemented a 4-week intervention consisting of five 1-min ZETx sessions twice daily. Their results confirmed that this resistance-based ZETx protocol effectively enhanced lower limb muscle strength (34). These collective findings robustly validate the efficacy of ZETx for improving musculoskeletal health in aging populations.

### 4.3 Cognitive function and mood regulation

As a non-pharmacological intervention, physical exercise has demonstrated significant advantages in promoting cognitive health. Current clinical and public health guidelines primarily emphasize sustained, structured physical activity. However, the implementation of such exercise regimens proves challenging for older adults populations with chronic conditions due to physical limitations, time constraints, or lack of access to appropriate facilities (35). In contrast, ZETx offers a more accessible alternative (10). ZETx effectively counteracts the detrimental cognitive effects of sedentary behavior—including reduced cerebral blood flow, elevated inflammatory markers, and diminished neuroplasticity—by incorporating multiple brief bouts of physical activity to break prolonged sitting (36). Research indicates that ZETx positively influences self-efficacy and mood regulation. Regular physical activity, particularly through jogging and walking, demonstrates significant associations with reduced depressive symptoms and improved emotional wellbeing (37, 38). Ribeiro et al. provided empirical evidence supporting the efficacy of ZETx in alleviating depressive symptoms (22). Furthermore, ZETx exhibits clinical value in sleep regulation. Through intermittent low-intensity contractions, it modulates core body temperature and regulates hypothalamic-pituitary-adrenal axis activity, thereby enhancing sleep quality (14). These multifaceted benefits position ZETx as a comprehensive behavioral intervention for cognitive and psychological health in vulnerable populations.

### 4.4 Improvement of metabolic parameters

Physical activity enhances insulin sensitivity, thereby stabilizing blood glucose levels. For chronic disease populations with limited physical capacity or disease-related restrictions, ZETx serves as an effective approach to improve metabolic parameters. Sedentary behavior contributes to abnormal glucose and lipid metabolism, whereas intermittent physical activity enhances insulin sensitivity by increasing muscle glycogen utilization and activating insulin-related signaling pathways in skeletal muscle (39, 40). Brief exercise sessions can reduce muscle glycogen levels by 20–30%, which helps lower blood glucose and improve insulin resistance (41). Short bouts of moderate-to-vigorous intensity activity can attenuate post-prandial spikes in blood glucose and insulin levels. As little as brief walking breaks every 30 min to interrupt prolonged sitting has been shown to reduce post-prandial glucose and insulin concentrations (42). ZETx activities such as stair climbing have demonstrated efficacy in lowering fasting blood glucose and improving post-prandial glycemic control in both healthy adults and sedentary individuals with impaired glucose tolerance. These benefits contribute to reduced diabetes risk, enhanced insulin sensitivity, and decreased fat mass (30). Current research consistently indicates that ZETx can improve lipid profiles (43). Hossein et al. found that brief ZETx sessions reduced post-prandial insulin and total non-esterified fatty acid levels in overweight or obese adults (44). Furthermore, ZETx has been shown to improve glycemic control in diabetic patients (45). These findings collectively support the metabolic benefits of ZETx across various populations and clinical contexts.

## 5 Analysis of acceptability and adherence to zero-time exercise interventions

ZETx demonstrates both favorable acceptability and adherence among chronic disease patients due to its low-threshold accessibility, high flexibility, and family-friendly characteristics, offering a feasible long-term health management solution (5, 20).

### 5.1 Acceptability

The low physical demand and simplicity of ZETx movements contribute to its high acceptability among chronic disease patients. By emphasizing brief, low-intensity activities during fragmented time periods, ZETx avoids excessive fatigue while accumulating health benefits through sustained practice—perfectly aligning with the exercise needs of chronic disease patients. The predominant focus on low-intensity activities (e.g., brisk walking, balance training) minimizes injury risks, particularly benefiting patients with multiple comorbidities. Integrating walking into commuting or household chores not only meets exercise requirements but also reduces psychological resistance to “exercise tasks,” thereby enhancing mental acceptance. Western et al. (33) reported an overall acceptability score of 4.6/5 among older adults patients with mild cognitive impairment participating in ZETx interventions, strongly supporting its potential as an acceptable exercise modality. Another 4-week resistance-based ZETx study in older adults demonstrated 75% participant acceptability, further confirming its feasibility in this population (20).

### 5.2 Feasibility and adherence

Regarding adherence, ZETx exhibits strong potential for long-term maintenance due to minimal time commitment, flexible scheduling, and seamless integration into daily life. A 12-week progressive home-based ZETx study revealed that 71% of participants adhered to the program, as the brief duration and convenience allowed completion without sacrificing other activities, with fixed daily timing further enhancing compliance (46). Research indicates superior exercise adherence in home environments (92% patient preference) (47), which aligns perfectly with ZETx implementation. Furthermore, family participation creates support networks that enhance exercise enjoyment and sustainability through social interaction, demonstrating unique advantages in improving adherence (48). Notably, self-reported adherence to ZETx interventions among cognitively impaired patients exceeds that of other exercise modalities (33), providing additional evidence for its applicability.

## 6 Current challenges and future perspectives

While ZETx demonstrates significant potential in chronic disease management, its implementation and widespread adoption face multiple challenges that require technological innovation and interdisciplinary collaboration to overcome.

### 6.1 Practical and patient-centered challenges

Despite its promise, the implementation of ZETx faces several practical challenges rooted in patient experiences and real-world applicability. A primary barrier is low perceived efficacy; some patients, particularly those accustomed to structured exercise, may doubt the health benefits of brief, sporadic activities, leading to low motivation and engagement (49). Furthermore, individuals with severe mobility limitations, chronic pain, or advanced disease may find even low-intensity ZETx movements physically challenging or may fear aggravating their condition without professional supervision (19). Environmental and social contexts also play a critical role; a lack of a supportive environment at home or work, or the absence of social cues and reminders, can hinder consistent practice (17). For instance, some patients in home-based studies reported forgetting to perform the exercises without external prompts (20), while others found integrating activity into certain daily routines (e.g., sedentary office work) more difficult than anticipated (44). These patient-centered insights highlight that the “ease” of ZETx is not universal and that successful implementation requires strategies to address perceived value, physical capability, and contextual barriers.

### 6.2 Urgent need for standardization of exercise prescription

The current lack of standardized protocols for exercise prescriptions poses a critical challenge, with significant variations in the definition of exercise intensity, frequency, and type across different medical institutions. This inconsistency not only affects the comparability of clinical studies but may also lead to reduced adherence or exercise-related risks due to inappropriate dosage. Exercise prescription, which tailors appropriate physical activity based on individual fitness levels and adherence capacity, aims to specify exercise duration, intensity, and precautions (50). Addressing this issue requires the development of intelligent assessment tools that utilize wearable devices for real-time monitoring of physiological indicators (e.g., heart rate, blood oxygen levels) combined with AI algorithms to dynamically adjust exercise regimens, thereby establishing a precision-based exercise prescription system grounded in individual health data (51).

### 6.3 Technological innovation to advance exercise interventions

Technological advancements offer promising solutions to current limitations. The integration of emerging technologies will expand the practical applications of exercise interventions (19). Virtual Reality (VR) and Augmented Reality (AR) technologies are increasingly being applied in the exercise domain. These immersive technologies can create engaging exercise environments for chronic disease patients by combining physical activity with virtual scenarios and gamification elements (e.g., VR-based stair-climbing simulations), thereby enhancing motivation and participation (52) (Table 2). However, several practical challenges need to be addressed for these technologies to be widely adopted: (1) the cost of VR and AR devices can be prohibitive for many patients, especially those with limited financial resources. Affordable and accessible technology is essential to ensure that these interventions are available to a broader population (53); (2) accessibility issues such as the need for technical support and the complexity of using these devices can limit their use, particularly among older adults patients. Ensuring that these technologies are user-friendly and require minimal technical knowledge is crucial (54); (3) digital literacy among older adults patients is a significant concern. Many older adults may not be familiar with the use of advanced technology, which can hinder their ability to benefit from VR and AR interventions (55). Future research should explore ways to overcome these challenges, such as developing cost-effective solutions, providing user-friendly interfaces, and offering training programs to enhance digital literacy.

**Table 2:** Key challenges and future directions for ZETx implementation.

**Challenge category**	**Specific gaps/barriers**	**Evidence-based future directions**
Practical and patient-centered	Low perceived efficacy of brief activities; Physical limitations/chronic pain in severe cases; Fear of worsening condition; Forgetting to exercise; Difficulty integrating into specific routines (e.g., sedentary office work); Lack of supportive environment/social cues.	Develop strategies to demonstrate and communicate efficacy (e.g., biofeedback); Tailor interventions for varying physical capabilities; Incorporate external prompts/reminders (e.g., apps, smart devices); Context-specific integration strategies; Foster supportive environments (e.g., family, workplace).
Standardization of prescription	Lack of standardized protocols; Significant variation in definitions of intensity, frequency, and type across studies/institutions; Potential for inappropriate dosage reducing adherence or increasing risk.	Develop intelligent assessment tools using wearable devices for real-time monitoring; Utilize AI algorithms to dynamically adjust regimens; Establish a precision-based exercise prescription system grounded in individual health data.
Technological innovation	High cost of VR/AR devices limiting accessibility; Complexity and need for technical support; Low digital literacy among key populations (e.g., older adults patients).	Develop cost-effective technological solutions; Design user-friendly interfaces requiring minimal technical knowledge; Provide training programs to enhance digital literacy; Explore accessible alternatives (e.g., smartphone apps).
Research and evidence base	Predominance of pilot trials with small sample sizes limiting generalizability; Lack of standardized ZETx definitions; Limited long-term data on sustained benefits and adherence.	Conduct large-scale, well-powered randomized controlled trials (RCTs) with standardized protocols; Establish consensus on ZETx definitions and measurement methods; Implement studies with long-term follow-up.
Multidisciplinary integration	Need for holistic intervention models beyond just exercise to address complex needs of chronic disease patients.	Develop multidisciplinary intervention models integrating expertise from exercise science, clinical medicine, nutrition, and psychology (e.g., combined “exercise-nutrition-psychological” programs).

### 6.4 Multidisciplinary collaboration for novel intervention models

Achieving widespread implementation of ZETx in chronic disease prevention and management requires the development of multidisciplinary intervention models. Interdisciplinary collaboration is paramount for establishing comprehensive and effective chronic disease interventions. Integrating expertise from exercise science, clinical medicine, nutrition, and psychology to design integrated “exercise-nutrition-psychological” intervention programs is particularly crucial. Research indicates that the synergistic effects of exercise with psychological modulation (e.g., flow state) and nutritional interventions (e.g., optimization of carbohydrate energy supply ratio) can significantly enhance chronic disease management outcomes (56).

### 6.5 Limitations and future directions

Despite the promising findings, this review acknowledges several limitations of current ZETx research. First, many studies are pilot trials or have small sample sizes, limiting the generalizability of results. Second, there is a lack of standardized definitions of ZETx; different studies operationalize “zero-time” or “fragmented” exercise in varying ways (e.g., differences in bout duration, intensity, or types of activities), making cross-study comparisons challenging. Third, long-term data is limited; most studies follow participants for a few weeks to months, and it remains unclear whether ZETx-induced health benefits are sustained over years or if adherence declines over time. These limitations highlight the need for future research to conduct large-scale, well-powered randomized controlled trials with standardized ZETx protocols and long-term follow-up. Establishing consensus on ZETx definitions and measurement methods will also be crucial to advance the field.

## 7 Conclusion

As an innovative strategy for chronic disease management, ZETx demonstrates distinct feasibility, flexibility, time efficiency, and convenience—characteristics that address key barriers to conventional exercise modalities for chronic disease patients. In summary, this review consolidates the conceptual framework, evidence base, and practical implications of ZETx, distinguishing it from broader discussions on physical activity by its specific operational principles and target population. It advances the field by critically synthesizing the available data on ZETx for chronic disease management, explicitly highlighting its theoretical underpinnings, its high acceptability, and the existing research limitations. By proposing a future roadmap that includes technological integration and the need for standardized prescriptions, this review provides a targeted and actionable resource for researchers, clinicians, and public health policymakers aiming to implement feasible exercise interventions for vulnerable populations. This approach provides physically inactive chronic disease patients with novel pathways to improve cardiovascular health, metabolic capacity, and muscular function. The demonstrated potential of ZETx in both empirical research and clinical practice warrants significant attention. Future directions should focus on exploring hybrid models integrating ZETx with traditional structured exercise programs. Policy-driven initiatives, particularly through community-based health promotion programs, will be crucial for widespread implementation. These developments are expected to expand and deepen the role of ZETx in chronic disease management, potentially transforming current practice paradigms.
